# Cross-analyzing addiction specialist and patient opinions and experiences about addictive disorder screening in primary care to identify interaction-related obstacles: a qualitative study

**DOI:** 10.1186/s13011-023-00522-5

**Published:** 2023-02-17

**Authors:** Maxime Pautrat, Caroline Renard, Vincent Riffault, David Ciolfi, Agathe Edeline, Hervé Breton, Paul Brunault, Jean Pierre Lebeau

**Affiliations:** 1 Faculty of Medicine, University of Tours, 10 Boulevard Tonnellé, 37000 Tours, France; 2 Department of General Practice, Tours Regional University Hospital, Tours, France; 3 UMR 1253, iBrain, University of Tours, Inserm, Tours, France; 4 Qualipsy EE 1901, University of Tours, Tours, France; 5 Équipe de Liaison et de Soins en Addictologie, CHRU de Tours, Service d’Addictologie Universitaire, Tours, France

**Keywords:** Addictive disorders, Addiction specialist, Substance use disorder, Mass screening, Primary health care, Early detection, Self-disclosure, Patient-centered approach, Interprofessional collaboration, Psychological barriers

## Abstract

**Background:**

Promptly identifying individuals with addictive disorders reduces mortality and morbidity and improves quality of life. Although screening in primary care with the Screening, Brief Intervention and Referral Treatment strategy has been recommended since 2008, it remains underutilized. This may be due to barriers including lack of time, patient reluctance or perhaps the timing and approach for discussing addiction with their patients.

**Objective:**

This study aims to explore and cross-analyze patient and addiction specialist experiences and opinions about early addictive disorder screening in primary care to identify interaction-related screening obstacles.

**Design and participants:**

Qualitative study with purposive maximum variation sampling among nine addiction specialists and eight individuals with addiction disorders conducted between April 2017 and November 2019 in Val-de-Loire, France.

**Main Measures:**

Using a grounded theory approach, verbatim data was collected from face-to-face interviews with addiction specialists and individuals with addiction disorders. These interviews explored their opinions and experiences with addiction screening in primary care. Initially, two independent investigators analyzed the coded verbatim according to the data triangulation principle. Secondly, convergences and divergences between addiction specialist and addict verbatim categories were identified, analyzed, and conceptualized.

**Key Results:**

Four main interaction-related obstacles to early addictive disorder screening in primary care were identified and conceptualized: the new concepts of shared self-censorship and the patient's personal red line, issues not addressed during consultations, and opposition between how physicians and patients would like to approach addictive disorder screening.

**Conclusions:**

To continue analysis of addictive disorder screening dynamics, further studies to examine the perspectives of all those involved in primary care are required. The information revealed from these studies will provide ideas to help patients and caregivers start discussing addiction and to help implement a collaborative team-based care approach.

**Trial registration:**

This study is registered with the *Commission Nationale de l’Informatique et des Libertés* (CNIL) under No. 2017–093.

**Supplementary Information:**

The online version contains supplementary material available at 10.1186/s13011-023-00522-5.

## Introduction

Addictive disorders are associated with significant morbidity, mortality, and high societal costs [1, 2]. People with substance use disorders involving alcohol, opioids, and cannabis risk premature death and disability, and have high levels of psychoactive, social, legal or violent consequences [3–5]. Also, non-substance addictive behaviors, such as gambling, share neurobiological and genetic similarities with substance use disorders and are also associated with high comorbidity. Substance use disorders and non-substance addictive behaviors are defined in the Diagnostic and Statistical Manual of Mental Disorders (DSM-5) [6].

When individuals with addictive disorders are identified early, the morbidity and mortality risk reduces and quality of life improves [7–9]. Yet the recommended Screening, Brief Intervention and Referral Treatment (SBIRT) strategy remains underutilized in primary care [10, 11]. Some barriers to screening for addictive disorder in primary care such as lack of time, feelings of ineffectiveness, and patient reluctance have been reported [12–17]. Furthermore, while some SBIRT stages have been widely evaluated [18–21], little is known about the screening stage. The SBIRT screening stage relies on specific validated questionnaires (AUDIT, DAST, CRAFFT 2.0) [22] which assesses the extent of an existing, known addictive behavior but do not identify people with early addictive behavior ranging from unhealthy use to addictive disorders. Yet, this initial, imperative, pre-screening step, is rarely performed in practice [23]. Also, the best timing and ways of discussing addiction in primary care remains unclear.

There is a need to understand the best approach to start a discussion about addictive behavior. On the one hand, patients may have feelings about their addiction which prevents them from seeking help, such as the fear of being considered an “outcast” [24]. On the other hand, addiction specialists have specialist knowledge to produce recommendations about screening for addictive disorders in primary care. To optimize interdisciplinarity care, it is therefore important to explore feelings, opinions, expectations, and experience about addictive disorder screening among both patients and addiction specialists. Previous studies have used a thematic approach to examine patient and physician opinions about addictive disorder screening in primary care. However, there is a need to use theoretical models to allow more in-depth analysis of individual attitudes and behavior change to further explore the known obstacles and facilitators [25].

Thus, this study will explore and cross-analyze patient and addiction specialist experiences and opinions about early addictive disorder screening in primary care to identify interaction-related screening obstacles.

## Methods

### Study design

This qualitative study recruited addiction specialists and patients with addictive disorders with purposive maximum variation sampling between April 2017 and November 2019. Data was collected, verbatims were coded independently according to the data triangulation principle, and ongoing inductive analysis and constant comparison was performed using a grounded theory approach [26].

### Population

In this study, addiction specialists were defined as physicians with extensive training and education in addiction treatment. Addiction specialists were recruited from primary care practices, hospitals and academic departments in the Centre-Val-de-Loire region, France. The first addiction specialist was contacted by phone then others were recruited using a snowball technique. Nine specialists aged between 38 and 62, of which seven were male and two females were involved. They worked in a hospital or outpatient setting or primary care and had varied specialties including addiction, psychiatry, emergency and general medicine and academia (Table 1). Eight patients with a DSM-5 confirmed addictive disorder were recruited from primary care, addiction specialists and hospital emergency units. Patients with tobacco addiction were excluded. There were three males and five females aged between 18 and 65. The living area was urban for five and rural or semirural for three. The treatment stage ranged from active to long-term abstinence. Four were employed, two unemployed, one student and one retired. Addictions included alcohol, cannabis, heroine, ecstasy, codeine, benzodiazepine, and gambling (Table 2).

**Table 1:** Participating addiction specialist characteristics

	**Participants *n* = 9**
**Age**	
<45 years	4
>45 years	5
**Gender**	
Male	7
Female	2
**Practice type**	
Hospital	5
Outpatient, CSAPA (Addiction Care, Support and Prevention Centers)	4
General practice	4
**Initial training**	
Psychiatry	3
Pulmonology	1
Intensive Care	1
**Current specialties**	
Specialist addiction liaison	3
General medicine	2
Psychiatry	4
Smoking (tobacco)	1
Emergency physician	2
Addictive behavior specialist	2
University	2

**Table 2:** Participating patient characteristics

		**Alcohol**	**Cannabis**	**Heroine**	**Ecstasy**	**Codeine**	**Benzodiazepine**	**Gambling**
**Female**	**Age**	55 years		54 years	18 years	26 years	57 years	
	**Living environment**	Urban		Urban	Urban	Rural	Semi-rural	
	**Professional activity**	Unemployed		Employed	Student	Employed	Employed	
	**Treatment stage**	Maintenance		Long-term abstinence	Active	Active	Active	
	**Type of monitoring**	Attending physician		Attending physician	Psychiatrist	Attending physician	Attending physician	
	**Interview duration**	55 min		62 min	53 min	79 min	67 min	
	**Number**	P2		P1	P4	P6	P8	
**Male**	**Age**	42 years	35 years					65 years
	**Living environment**	Urban	Rural					Urban
	**Professional activity**	Unemployed	Employed					Retired
	**Treatment stage**	Maintenance	Active					Maintenance
	**Type of monitoring**	Attending physician	Addiction center					Addiction center
	**Interview duration**	85 min	99 min					97 min
	**Number**	P5	P3					P7

All participants were informed about the study and its objectives and provided informed consent (Additional files 1 and 2).

### Data collection

Interviews with addiction specialists were conducted in their workplace. Interviews, guided with a specifically designed guide to explore addiction specialist experiences and opinions about addiction screening in primary care lasted between 31 and 79 min. The guide was modified throughout the data collection process to include new concepts that emerged during the simultaneous analysis (Additional file 3).

Interviews with patients with addictive disorders were conducted in their usual care setting Interviews were guided with the McGill Illness Narrative Interview (MINI) guide [27, 28] (Additional file 4) and lasted between 53 and 99 min. This guide explores life trajectories through narrative, prototypical and causal biographical reasoning.

Two male general practitioners (GP) who had received interview training, conducted all interviews. The participants did not know the interviewers before the study and no-one else was present during the interviews. The interviewers informed each participant about their role as a GP and that this research was for their theses.

All interviews were audio recorded and transcribed. All verbatim was coded to anonymize participant identity using P1-8 for the patients and AS1-9 for addiction specialists. No interviews were repeated, and no notes were made during the interviews. Transcriptions were not shared with participants.

### Data analysis

The verbatim from each interview was coded according to the data triangulation procedure as follow: firstly, two analysts performed the initial coding using a coding book where necessary and discrepancies were resolved by discussion with arbitration from a third analyst if needed, until data sufficiency. Secondly, categories were built from the initial coding book using the same triangulation procedure. Grounded theory was used for the specialist codes and a narrative approach was used for the patients with addictions. Thirdly, all the initial verbatim was reanalyzed and categories were compared and redefined according to the grounded theory approach. Convergences and divergences were identified and analyzed to create an explanatory model. The software package QSR NVivo11® facilitated category analysis and systematic comparison.

To ensure the results were relevant, two addiction specialists, a narrative research specialist and four GPs provided their opinions about this analysis. All participants were invited to provide feedback on the verbatim from their interviews and study findings.

### Ethical aspects

The *Espace de réflexion éthique région Centre* approved this study (N°2017–059, le 09/01/18) (Appendix 3). This study is registered with the *Commission nationale de l’informatique et des libertés* (CNIL) under N°2017–093 (Appendix 4). The research was reported in accordance with the COREQ/SRQR guidelines.

## Results

In total, eight patients and nine addiction specialists were interviewed. No addiction specialists or patients refused to participate in the study.

### A shared self-censorship

#### The silence of shame among patients...

Many patients felt stigmatized: *“we are quickly categorized. As soon as something happens in my neighborhood, they say ‘Ah, addicts!’. It’s disdain, marginalization”* [P1]. Other patients expressed self-derogation: *“I sometimes look at myself, at my dirty clothes and unwashed hair and say ‘well, you look dreadful today, you’re so pale!”* [P1]. Furthermore, the addictive patient verbatim was filled with shame: *“Of course there is shame, because you know that you will be asked questions about your private life”* [P7].

Patients isolated themselves to avoid this shame and stigmatization: *“I drank in secret, even in my parent’s house. I found myself in awful shape”* [P2], *“I was in a dreadful state. I’ve never been like that before. I was in the middle of the city, and I couldn’t even articulate two words. Babbling is very degrading. But finding myself with my arms tied in a bed is also very degrading”* [P4].

This feeling of isolation reinforced the feelings of loneliness and the difficulty patients had talking about it: *“cannabis is still very controversial”* [P3], *“these questions involve our private lives, we don’t all want to go out and reveal our private lives”* [P8]. When patients talk to physicians, they described how the feeling of shame led to self-censorship: *“When I’m with the doctor, she seems sweet, but when I wanted to talk to her about it, I withdrew, I didn't dare”* [P6].

Addiction specialists described this patient censorship as a major barrier to detecting addiction. Patients are often ashamed of their substance use and cannot talk freely with their physician about it meaning they are *“unable to seek help for a long time”* [AS7]*.* Furthermore, many patients described self-stigmatization further preventing them from talking openly. They feel people look at them when they are out in public maintaining a *“form of shame, or moral judgment”* [P3]*.*

 ... **echoes the silence of embarrassed physicians**

Many addiction specialists mentioned that physicians fear broaching the subject resulting in self-censorship of the physician. *“Physicians are afraid of the patient’s reaction. They fear causing shame and guilt which would end the discussion when in fact, it often starts it”* [AS2]*.* Furthermore, specialists discussed that physicians are sometimes afraid *“of not knowing how to evaluate the addiction, of not knowing what to do, and of not knowing who to refer the patient to”* [AS1]. They suspected that GPs rationalize addictive disorders, particularly non-substance addictive behaviors such as gambling: *“Physicians can feel like it’s not their problem, and when you ask the general population, they feel it’s a choice, not a disease”* [AS5]*.* Furthermore, some GPs may feel that the substance the patient is addicted to is the *“last thing they have left, so maybe you shouldn’t take it away”* [AS7].

### The obvious versus the red line

#### The physician’s need for an obvious semiology...

Many addiction specialists reported situations where addiction-related health issues are only identified when the clinical presentation is obvious*: “when there are signs of withdrawal, dependency or pharmacological signs, identification is quite easy”* [AS5]*.* This means that “*identification often only occurs when patients are scared of these symptoms, or during an obvious withdrawal”* [AS5]. Most specialists felt that the diagnosis could and should be made when patients complain of non-specific disorders such as *“sleep disorders”* [AS3] or *“chronic pain”* [AS6]. It can even happen during *“an emergency situation such as a patient passing out on a public road, being found by an ambulance and then hospitalized”* [AS7].

However, most addiction specialists agreed that the accumulation of addiction-related problems was not always enough to encourage patients to seek help; “*even when it’s obvious to their relatives, it’s not necessarily so for the patient”* [AS5].

… **and the patient’s personal red line**

Many patients explained that they were aware of the damage caused by their addiction: *“Dependency sets in when you realize that something is missing, that you can no longer go to the supermarket without buying any alcohol. It’s also in my body; nausea, vomiting, alcohol poisoning”* [P5]*.* They also knew that their relatives were aware of their addiction: *“My relatives realized when the illness was already well established. It reaches a point when there’s no use hiding it anymore! My parents told me: ‘you have to do something; you can’t continue like this anymore’. But I already knew that”* [P3]*.*

Most addiction specialists described an unpredictable delay between diagnosis and *“something clicking inside the patient”* [AS5] leading to change. This turning point (the red line) appeared to be patient-specific: *“Nobody pushed, forced, or made me do it. It was me; it came from me”* [P1]*. “You can’t force someone to change, it’s not possible”* [P6]*.* The red line could be a point of no return such as a physical emergency: *“I said to myself ‘I can’t stay like this; I’m going to die’”* [P3] or a psychological event: *“Something clicked and I realized I’d lost everything. The distress was unbearable”* [P1]. It could also be a change in circumstances requiring substance-use cessation, particularly pregnancy: *“Something clicked because we were trying to have a baby”* [P6] or *“I was afraid for the baby and told myself ‘you can’t go on like this until you die! You must do something!’ I had a moment of clarity”* [P6].

### Patient reluctance to disclose and physician failure to listen

Some patients felt their physician lacked consideration: *“He knew it, he wasn’t stupid, he must have realized that I wasn’t in great shape! But he never asked me about anything, I think he didn’t really care!”* [P1]*.* Others described feeling left alone to deal with their condition: *“I told them about it, but nothing happened”* [P6]. Some thought that they didn’t need to say it: *“People’s discomfort can be seen, even when they try to hide it”* [P8] and seemed disappointed when the physician’s reaction was not what they had expected: *“I told him that I have a problem with alcohol. He said: ‘It’s not that serious. Just take the Esperal®’. It was not what I needed, I needed much, much more! Things didn’t turn out well after that”* [P5]*.*

Some addiction specialists also shared this impression that some physicians lack investment: *“Patients feel that their physician didn’t want to take care of them. Saying ‘You’ll come back when you’re ready’ is just terrible”* [AS8]. Most specialists highlighted the importance of taking the patient’s reluctance to talk into consideration: *“When patients manage to talk about it, there are physicians who say, ‘well now you have to stop drinking, we’re going to do a withdrawal, I’ll write you a prescription’. If the patient seems reluctant or starts to be a little scared by all this, the physician stops and says ‘Well, come back when you’re ready!’ He’ll be back 20 years later as an emergency with decompensated cirrhosis”* [AS8]. Many addiction specialists also regretted the fact that physicians sometimes miss the opportunity to start treatment, which can mean the patient finds it harder to change.

### Should we put away validated tools and take off our white coat?

#### A consistent approach based on standardized questionnaires...

Many addiction specialists felt it was necessary to use standardized tools:* "Using a consistent approach, or consistent sentences, could eventually unlock some situations and allow the patient to finally say ‘yes, I am like all these people in fact”* [AS5]*.* This approach aims to limit stigmatization and facilitate disclosure: *“In my opinion, it is better to suggest* it *[the potential addictive disorder] to everyone, to destigmatize it”* [AS8], *“These things are routinely asked as soon as a patient arrives”* [AS4], *“Self-reporting tools also exist. People fill them out by themselves, there is no need for a professional’s help, and they can therefore give a fairly accurate picture of what people consume”* [AS8], “*We really have to try to standardize the questions”* [AS5].

**… but open our ears with empathy and build trusting relationships**

Most patients expressed a desire for an empathetic and sincere approach to build trust: *“I have disclosed to people I trust. She was very attentive, very understanding”* [P6], *“I didn't want to reveal myself because I didn't trust them enough”* [P3]. Many patients sought “*Listening without judging, without having preconceived ideas. I think there are as many situations as there are people”* [P1]. *“It's all about listening! It takes more than one consultation!”* [P8].

Most patients reported their need for human contact: *“I think the human side is needed to detect this kind of thing”* [P1], *“Having a good, understanding doctor is the only possible way I am going to heal”* [P7]. Patients felt that the physician’s experience with substance use disorders was not important: *“I think it's something you can't learn”* [P8], *“Even if your doctor doesn’t know much, he can open some books, learn a bit and that’s fine”* [P5]. Some even mentioned the desire to change how consultations are conducted:* “maybe not seeing the doctor in a medical context, but over a coffee. With a cigarette, maybe in a coffee shop”* [P8].

## Discussion

**Fig. 1:**
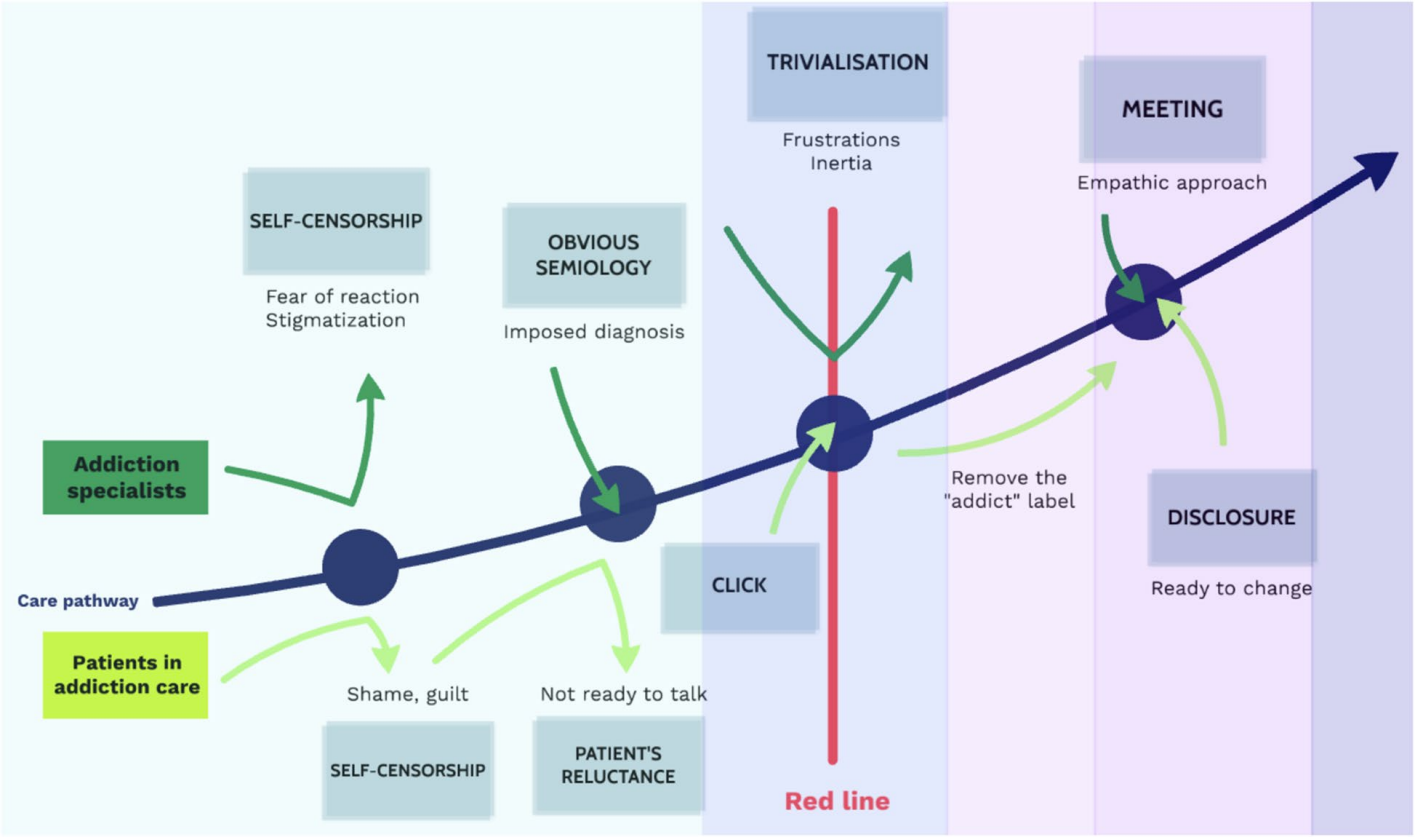
Theoretical model of screening for addictive disorders

To our knowledge, this is the first study to use a combination of grounded theory, a narrative approach and cross-analysis to examine the opinions of addiction specialists and patients with addictions. Using grounded theory enabled in-depth analysis of addiction specialist opinions going beyond the known obstacles such as GP lack of time or knowledge. The narrative approach enabled a chronological addiction trajectory to be elaborated and the obstacles and expectations to be presented along it (Fig. 1). Cross-analysis highlighted the similarities and differences in opinions between addiction specialists and patients.

The study reveals four main patient/physician interaction-related obstacles to early addictive disorder screening in primary care: the new concepts of shared self-censorship and the patient's personal red line, issues unaddressed during consultations, and opposition between how physicians and patients would like to approach addictive disorder screening.

### Removing self-censorship

This study reveals shame, low self-esteem and a sense of worthlessness in patient discourse which has been described in previous studies [29–31]. The double stigmatization resulting from the caregivers’ views and the patients’ negative view of themselves, explains why patients are reluctant to disclose their problem. “*Most patients won’t bring it up themselves*” [25].

Physicians are also reluctant to raise the subject of addictions [31, 32]. Addiction specialists suggest that some physicians avoid the subject since they believe the drive for change comes from the patient [33].

For the first time, cross-analysis of patient and addiction specialist verbatim revealed the new concept of shared self-censorship with mutual avoidance. Avoidance has long been described in anxiety disorders and is a factor in maintaining anxiety. In order to control their anxiety level, people with anxiety disorders often avoid situations associated with stress and learned helplessness responses [34]. In this current study, the anxiety-provoking situation is starting a conversation about addictive disorders [35].

### The patients’ personal red line

Since addictive disorders are associated with an accumulation of co-morbidities, the need for physician involvement increases [36]. However, our study reveals that these accumulated addiction-related co-morbidities do not lead patients to start talking about their addiction. Existing literature discusses “teachable moments”, such as an overdose, which can be used to combine acute treatment with referral to an addiction specialist [25, 31]. This implies that this turning point is a passive moment, something that the physician can utilize to begin specialist addiction therapy.

In contrast, our study reveals that each patient has their own personal red line. This turning point is patient-specific and is triggered by an event in the patient’s life leading to an active decision to change and start talking. Relatives and physicians cannot trigger a person to cross their personal red line but patient verbatim suggests that they want their physician to gently encourage them to change. To our knowledge, this is the first study to reveal that this turning point is truly personal and different for everyone.

### The importance of what is and isn’t said

Our results suggest that when physicians avoid asking about problematic substance use, this can lead the patient to trivialize the problem, *“if you don’t talk about it, there is no problem”* [AS7]. This echoes the “not sick enough” concept known in other behavioral disorders [37]. Furthermore, the patient’s resistance to discuss their addiction until they cross their own red line can frustrate physicians [38, 39]: *“when you ask the question but don’t get an answer, it’s easy to get impatient. If after asking several times you still have no response, you stop asking”* [AS6]. When the patient finally starts to talk, they are sometimes disappointed by their physician’s response, feeling that they *“don’t care”* [P1] which reinforces the disorder and can lead to initiation of care inertia. Care inertia results from the interaction of different factors, mainly physician-related factors such as reactive rather than proactive care, and patient-related factors including poor communication and low health literacy [40].

### Stepping out of the comfort zone

Addiction specialists highlighted the importance of a standardized medical approach to prevent preconceptions in primary care [41] often through the use of questionnaires as per current health recommendations [42–44]. Some formulations have been suggested [45], without providing advice on how to start the discussion. This can create an obstacle to early screening.

Patients described social exclusion and belonging to a stigmatized social group [31] labelled as *addicts.* Removing this label requires a change of social circle but, *“it’s hard to get a new social life afterwards”* [P4]. The physician can become the first social contact in this process making consultations a transitional tool for dynamic change. A patient-centered approach is essential to ensure physicians can adapt to each patient’s level of understanding, and their motivation to disclose and change.

Patient-centered care is widely recommended as it prioritizes the unique needs of each patient and encourages a more equal balance of power between physicians and patients. In substance use disorder treatment, therapeutic alliance including empathy and non-judgment is the most important principle of patient-centered care, followed by shared decision-making, and individualized, holistic care [46].

### How do we meet our patients’ expectations?

Recent thematic analysis studies brought additional understanding of substance use screening through analysis of patient and physician opinions [25, 47]. These studies discussed the importance of regular annual screening within a limited amount of time, the benefit of using self-reported questionnaires to limit discomfort in face-to-face consultations, and physician knowledge deficit. Our study, which adopted a theoretical analysis approach based on grounded theory, enables a more in-depth exploration of these themes raised using a thematic approach.

Furthermore, for the first time, cross-analysis of addiction specialist and patient opinions has highlighted the gap between what addiction specialists believe they should be doing to meet patient expectations and what patients really expect from their caregivers. Patients described wanting an empathetic, non-judgmental, and caring physician to help them break their self-censorship. They would like the physician to make the first move, ask them when they are ready to start talking, and listen to them when they are ready to talk. However, they fear that their physician will stigmatize them and hinder their motivation to talk. This demonstrates the importance of physicians remembering patient expectations during consultations. In contrast, physicians tend to use a psychometric, technical identification approach using tests to identify disorder severity. This difference in expectations can create an obstacle to early screening.

The new patient-specific red line concept should also be considered when trying to identify addictive disorders. Each patient has their own red line and may need encouragement to talk, but they do not expect physicians to be so passive as to say, *“You drink, come back when you are ready to stop”* [AS8]. In addition, patients in our study described the importance of human contact and having an understanding physician. Interestingly, they also emphasized that the physician’s experience with substance use disorder was not important which opposes findings in existing literature stating that a lack of knowledge could be a potential obstacle [47]. Approaching the issue with an empathetic, patient-centered approach which is not overly standardized may facilitate discussion. Furthermore, providing a friendly, trusting, relaxed and safe environment may encourage patients to cross their red line.

The reluctance to start talking has been discussed at length in the literature but our results provide more detail on this concept. Not only do patients have a certain reluctance to talk which reinforces the caregiver’s frustration, but caregivers trivialize the addictive disorder which reinforces the trivialization of the disorder that the patient already perceives. Frustration and stigmatization are known to negatively impact physician–patient relationships [33, 38, 39, 41]. Similarly, it is possible that trivialization may impact the therapeutic bond. These reflections, illustrated by our results, reveal the association between omission and trivialization, confession and stigmatization, concealment and frustration, and inattention and deception, which help to clarify the patient-physician relationship surrounding addictive disorders.

New technologies and e-health are starting to facilitate the early screening process making the physician’s job easier. A tool to help identify tobacco and alcohol use disorders has already been developed using Artificial Intelligence [48] and an e-SBIRT version has been developed [49]. However, whether these innovations make it any easier for the patient remains to be seen. 

### Strengths and limitations

This study has several main strengths. Grounded theory analysis enabled concepts to be identified from cross-analyzing specialist and patient experiences [26, 50]. Furthermore, the strictly inductive analysis and the data triangulation limited any influence from investigator subjectivity. The COREQ criteria were respected throughout the study [51].

The purposive sampling provided the required diversity in the patient population. However, there are very few addiction specialists so obtaining maximum variation in this population was difficult. The nine included addiction specialists do however represent around 80% of addiction specialists in the Centre-Val-de-Loire region, France making our sample an adequate representation.

This type of analysis is subject to the typical limitations including interpretation and confirmation bias [52]. However, multidisciplinary collaboration (psychiatrists, addiction specialists, GPs, sociologists) should have enriched data analysis and limited this bias. Member-checking can also reduce confirmation bias. Unfortunately, despite being given the opportunity to do so, none of the participants provided feedback on the verbatim from their interview or study results.

Patients with tobacco addiction were not included in this study as there is less stigma surrounding this addictive disorder, screening often occurs through cardiovascular prevention and monitoring, there are no psychoactive consequences and there is a lower level of social, legal, or violent consequences associated with this substance.

### Perspectives

The concepts revealed in this study and elements in the literature have been used to create a theoretical model of addictive disorder screening practice (Fig. 1). To continue the analysis of addictive disorder screening dynamics started in this study and illustrated in the theoretical model, further studies are required to examine the perspectives of those involved in primary care including GPs, patients only monitored in primary care, and paramedical professionals. For example, it has been shown that different healthcare professionals have different strengths and weaknesses when it comes to SBIRT training and implementation [53]. Until the theoretical model has been completed with this information it is difficult to suggest solutions to the barriers revealed in this study. However, completing the theoretical model will provide some ideas to help patients and caregivers open the door to a conversation that is difficult to start and help implement a collaborative team-based care approach.

## Conclusion

The novel study design combining grounded theory, a narrative approach, and cross-analysis enabled in-depth exploration and comparison of patient and addiction specialist opinions to be performed and a theoretical model of the chronological addiction trajectory presenting obstacles and expectations to be created. Four interaction-related obstacles to early addictive disorder screening were revealed: the new concepts of shared self-censorship and the patient's own red line, issues not addressed during consultations, and opposition between how physicians and patients would like to approach addictive disorder screening. To continue analysis of addictive disorder screening dynamics and complete the theoretical model, further studies to examine the perspectives of all those involved in primary care are required. The information revealed from these studies will provide ideas to help patients and caregivers start discussing addiction and to help implement a collaborative team-based care approach.

## Supplementary Information

**Additional file 1.**

**Additional file 2.**

**Additional file 3.**

**Additional file 4.**

## Data Availability

The datasets used and analysed during the current study are available from the corresponding author on reasonable request.
