# Supplementary material for: Cross-analyzing addiction specialist and patient opinions and experiences about addictive disorder screening in primary care to identify interaction-related obstacles: a qualitative study
Source: Subst Abuse Treat Prev Policy. 2023 Feb 17;18:12. doi: 10.1186/s13011-023-00522-5 (PMC9938560; doi:10.1186/s13011-023-00522-5)
Supplement: Supplementary file 2 — Additional file 2. [file 13011_2023_522_MOESM2_ESM.pdf]

RESEARCH NEWSLETTER
Version No. x from xx/xx/ xxxx

Acronym:
PAPRICA study: Problematic use and Addiction in Primary Care

Research Sponsor:
Tours University Hospital - 2, boulevard Tonnellé 37044 Tours Cedex 9

Coordinating investigator:
Dr PAUTRAT Maxime
General Practitioner – Head of General Medicine Clinic of
Tours University Hospital - 2, boulevard Arbor 37044 Cedex Towers 9
Telephone: 02 18 08 20 00

Dear ,

You have been invited to participate in a study organized by Tours University Hospital called *PAPRICA*.

This so-called non-interventional research does not involve any risk or constraint for you. Nevertheless, in the absence of opposition, processing of your health data may be implemented.

Take the time to read the information contained in this document and ask all the questions you think will help you understand it. You can take the time necessary to decide if you wish to object to the data concerning you being used for this research.

WHAT IS THE OBJECTIVE OF THIS STUDY?

The objective is to explore addiction specialist opinions about identifying problematic consumption, behavior and polydependency to find any obstacles to this identification, compare your expectations with those of patients, and see if improvements are possible in coordination with other caregivers.

WHAT WILL HAPPEN IF I PARTICIPATE IN THE RESEARCH?

If you do not object to participating in this research, the data concerning you will be collected and processed to meet the objective mentioned above.

Your participation will last for the duration of this interview and there will be no additional visits.

CAN I WITHDRAW MY PARTICIPATION?

Your participation is entirely voluntary. You are therefore free to change your mind at any time and to object, without having to justify yourself, to the processing of your data in the context of this research.

In this case, you must inform the research coordinator or the doctor who suggested that you take part. They will then communicate your opposition to the sponsor.

WILL MY PARTICIPATION REMAIN CONFIDENTIAL?

As part of this research, computer processing of the data collected will be implemented to allow analysis of the research results. This data may also, under conditions ensuring their confidentiality, be transmitted to the sponsor or to persons acting on its behalf. This data will therefore be identified by a code and the initials of your surname and first name.

In accordance with the provisions of the law relating to data processing, files and freedoms, you have a right to access and rectify computerized data concerning you at any time (law no. 2004-801 of August 6, 2004 modifying Law No. 78-17 of January 6, 1978 relating to data processing, files and freedoms). You also have the right to oppose the transmission and processing of data covered by professional secrecy that may be used in the context of this research. You can access directly or through the sponsor all the verbatim statements of the interview that we will do with you during this study.

WHO APPROVED THE RESEARCH?

Pursuant to the provisions of Article L1121-4 of the Public Health Code, the terms of this research were submitted to a Committee for the Protection of Persons (CPP) whose mission is to verify the conditions required for the protection and respect of your rights.

WHO CAN I CONTACT IF I HAVE ANY QUESTIONS?

The doctor who proposed this research to you is at your disposal to provide you with any additional information. If you wish, you can contact the research coordinator directly: Dr PAUTRAT (02 18 08 20 00)

To be completed by the investigator (doctor or qualified person)

Investigator surname and first name: ....

Telephone number: |___|___|-|___|___|-|___|___|-|___|___|-|___|___|

Surname and first name of the person participating in the research: .... ……….

Date of issue of the information: ___ / ___ / ______ Opposition expressed: ☐**Yes** ☐**No**

Investigator signature:

To be completed in duplicate: the 1st copy is to be kept by the investigator, the 2nd by the participant.

OBJECTION FORM
TO THE USE OF HEALTH DATA FOR RESEARCH
Version No. x from xx/xx/ xxxx

Study Acronym:

PAPRICA study: Problematic use and Addiction in Primary Care

Research Sponsor:

Tours University Hospital - 2, boulevard Tonnellé 37044 Tours Cedex 9

Coordinating investigator:
Dr PAUTRAT Maxime
General Practitioner – Head of General Medicine Clinic of
Tours University Hospital - 2, boulevard Arbor 37044 Cedex Towers 9
Telephone: 02 18 08 20 00

To be completed by the person who agrees to the research only in case of opposition

Contact details of the person agreeing to the research:

Surname: ……………………………………………………………………

First name: ……………………………………………………………………

☐ **I object to the use of my health data in this research.**

☐ **If applicable, I object to the use of all data collected previously.**

You can reconsider your decision at any time, all you have to do is inform the research coordinator or the doctor who asked you to take part .

Date: ___ / ___ / ______ *Signature:*

After completing this document, please give it to the doctor (or the qualified person depending on the type of study) who suggested you participate or directly to the research coordinator.
