# Supplementary material for: Cross-analyzing addiction specialist and patient opinions and experiences about addictive disorder screening in primary care to identify interaction-related obstacles: a qualitative study
Source: Subst Abuse Treat Prev Policy. 2023 Feb 17;18:12. doi: 10.1186/s13011-023-00522-5 (PMC9938560; doi:10.1186/s13011-023-00522-5)
Supplement: Supplementary file 3 — Additional file 3. [file 13011_2023_522_MOESM3_ESM.pdf]

Interview guide – addiction specialist

My name is (name of intern). I am a general medicine intern at Tours. My thesis focuses on the realities of identifying problematic behaviors and substance use, and addictions. It is part of a larger project called PAPRICA (Problematic use and Addiction in Primary Care), the aim of which is to identify the problems encountered in identifying problematic addictions and to provide solutions.
This is a qualitative study using interviews with addiction specialists.

This interview is recorded and anonymous. It will then be transcribed in writing to be analyzed using the grounded theory method.

Interview guide :
No questions, but words, reflections, themes from the literature review
Always ANCHORING on the theme and expressing the CONVICTIONS
Naive speech, question as vaguely as possible
—> **And always add: why?**

Icebreaker question :
- Tell me the story of the last patient you saw.
- How did he get to your practice?
- Is it always like that? Why?

Missed sightings:
- Tell me the story of an addiction screening that failed.
- Why do you think it failed?
- What could have been improved?

Successful referrals :
- Now tell me the story of a screening that went well.
- What are the positive elements of this situation?
- What brought the patient to come to see the doctor the first time?

General medicine:
- What do you think of screening in general medicine?
- What do you expect?
- What obstacles do you see?
- In your opinion, what should be done to improve how general practitioners identify addictions?
