# Supplementary material for: Cross-analyzing addiction specialist and patient opinions and experiences about addictive disorder screening in primary care to identify interaction-related obstacles: a qualitative study
Source: Subst Abuse Treat Prev Policy. 2023 Feb 17;18:12. doi: 10.1186/s13011-023-00522-5 (PMC9938560; doi:10.1186/s13011-023-00522-5)
Supplement: Supplementary file 4 — Additional file 4. [file 13011_2023_522_MOESM4_ESM.pdf]

Interview guide

Based on the articles:
1) Groleau , Danielle, Allan Young, and Laurence J. Kirmayer . "The McGill Illness Narrative Interview (MINI): An Interview Schedule to Elicit Meanings and Modes of Reasoning Related to Illness Experience." Transcultural Psychiatry 43, no. 4 (December 1, 2006): 671–91. doi: 10.1177/1363461506070796.
2) "Presentation and use of an interview guide in narrative medicine: the MINI." Thubert Clémence, Pascal Cathébras , Jean Monnet University (Saint-Étienne), and Jacques Lisfranc Faculty of Medicine . [exercise 2016;124:85-93], 2016.

Firstly:

- Introduce yourself: I am 'your name', intern in the 9th year of medicine (add "that is to say that I am a doctor but not yet a doctor" if necessary) and I am doing research work for my thesis on "The identification of problematic drug use and addictions »
- Discuss with the interviewee the study objectives (problematic uses and addictions) and the terms used to ensure that their understanding is shared.
- Inform the interviewee that the interview is confidential, will be transcribed and that their identity will not be revealed.
- Sign consent

1. Part One: Initial Information about the Illness

1. When did your addiction problems start? [Continue as long as possible by simply asking "What happened next? And then?"]

2. We would like to know more about your experience. When did you realize you had addiction issues?

3. Can you tell me what happened when you started having your addiction problems?

4. Did something else happen? [Continue as long as necessary to bring out *(to express...)* the events and experiences by asking "What else happened?"]

5. If you went to see someone to help you such as a caregiver, tell us how the visit went and what happened next.

6. If you have seen a doctor, tell us how it went (leave the field open to the different methods of meeting: consultation / hospitalization / going to the emergency department) and what happened next?

   6.1. Have you had any tests or undergone treatment for your addiction problem?
   [The relevance of this question depends on the health condition]

2. Part Two: Prototypical Narrative

7. In the past, have you had a health problem that you consider similar to your current addiction problems? [If the answer is yes, go to question 8]

8. Why was this health issue like your current addiction issues? Why was it different?

9. Has anyone in your family ever had the same addiction problems as you? [If the answer is yes, ask question 10]

10. How is your health problem similar or different from that of this other person?

11. Do you have a friend or colleague around you who has had the same type of health problem as you? [If the answer is yes, ask question 12]

12. How was this health problem different or similar to your health problem?

13. Have you ever seen on television, heard on the radio, or read in magazines, books or the internet about someone who has had the same problem as you? [if the answer is yes, go to question 14]

14. How is your health problem similar or different from that of this other person?

3. Third part: Narrative of an explanatory model

15. Do you have another word, expression or another way of designating your addiction problems?

16. Why do you think you had addiction problems? What do you think caused it?
[list main/primary causes]

17. Are there other causes that for you have played a role? [list secondary causes]

18. Why did your addiction problems start then? What happened to your body that could explain your addiction problems?

19. Has something happened in your family, at work or with your friends, or acquaintances… that could explain your addiction problems? Or, Has something happened in your personal, family or professional life that could explain your addiction problems? [if the answer is yes, go to question 20]

20. Can you explain to me how this explains your addiction problems?

21. Did you think you might have a drug problem?

22. What do you think about drug problems? What does this mean for you?

23. What usually happens to people who have drug problems?

24. What is the best treatment for people who have *(these)* drug problems?

25. How do people react to someone who has drug problems?

26. Who do you know who has had drug problems?

27. How are your addiction problems similar or different from this other person?

28. Are your addiction problems related in any way to specific events in your life?

29. Can you tell me more about these events and how are they related to your addiction problems?

4. Part Four: Aids and Response to Treatment *(Services and Response to Treatment)*

30. When you visited the doctor (or other broader caregiver), what did they tell you about your addiction problems, what did they call them?

31. Has your doctor (or other broader caregiver) given you any treatment, medication, or advice to follow regarding your addiction problem? [list them all]

32. How's it going ? [repeat questions 33 through 36 as needed for each advice, medication and treatment.]

33. Can you follow this treatment (or advice or drug treatment)?

34. What makes this treatment work?

35. What makes this treatment difficult to follow or does not work well?

36. What treatment did you expect to receive for your addiction problems that you did not receive*?*

37. What other care, treatment, help or care have you sought?

38. What other support, treatment, help or care would you like to receive?

5. Fifth part: impact on life

39. How have your addiction problems changed the way you live?

40. How have your addiction issues changed the way you feel or see yourself?

41. How have your addiction issues changed your outlook on life in general?

42. How have your addiction issues changed the way others see you?

43. What helped you get through this time in your life?

44. How did your family or friends help you through this time in your life?

45. How has your spiritual life, faith or religious practice *(religion)* helped you through this difficult time in your life?

46. Do you have anything else to add?
